# Evolutionary Game-Theoretic Approach to the Population Dynamics of Early Replicators

**DOI:** 10.3390/life14091064

**Published:** 2024-08-25

**Authors:** Matheus S. Mariano, José F. Fontanari

**Affiliations:** Instituto de Física de São Carlos, Universidade de São Paulo, Caixa Postal 369, São Carlos 13560-970, SP, Brazil; matheussmariano@usp.br

**Keywords:** prebiotic evolution, replicator equation, stochastic simulations, enzyme production, public goods

## Abstract

The population dynamics of early replicators has revealed numerous puzzles, highlighting the difficulty of transitioning from simple template-directed replicating molecules to complex biological systems. The resolution of these puzzles has set the research agenda on prebiotic evolution since the seminal works of Manfred Eigen in the 1970s. Here, we study the effects of demographic noise on the population dynamics of template-directed (non-enzymatic) and protein-mediated (enzymatic) replicators. We borrow stochastic algorithms from evolutionary game theory to simulate finite populations of two types of replicators. These algorithms recover the replicator equation framework in the infinite population limit. For large but finite populations, we use finite-size scaling to determine the probability of fixation and the mean time to fixation near a threshold that delimits the regions of dominance of each replicator type. Since enzyme-producing replicators cannot evolve in a well-mixed population containing replicators that benefit from the enzyme but do not encode it, we study the evolution of enzyme-producing replicators in a finite population structured in temporarily formed random groups of fixed size *n*. We argue that this problem is identical to the weak-altruism version of the *n*-player prisoner’s dilemma, and show that the threshold is given by the condition that the reward for altruistic behavior is equal to its cost.

## 1. Introduction

The issue of the evolution of cooperation [1] officially entered the field of prebiotic or chemical evolution when Maynard Smith [2] noted that providing catalytic support in a molecular catalytic feedback network, such as the hypercycle [3,4], can be seen as altruistic behavior. Since the usual framework for studying the evolution of cooperation is evolutionary game theory [5], there has been a fruitful intersection between game theory and prebiotic evolution [6,7,8], which has spilled over into viral evolution [9,10], as viruses are currently the best natural realization of a replicator. A replicator refers to a hypothetical entity that has the ability to make copies of itself through some form of replication process.

The study of the population dynamics of error-prone replicators has revealed (or created) a number of puzzles that make it difficult to explain the transition from simple template-directed replicating molecules to more complex biological systems [11]. For example, an enzymatic molecular replicator must be long enough to store information that codes for an enzyme (replicase), but a long replicator cannot be accurately copied without the assistance of a replicase, leading to a “chicken-and-egg” puzzle [3,12]. Splitting the information for encoding the enzyme into different short replicators does not work because different replicators cannot coexist in a purely competitive scenario [13]. Even in a scenario of perfect template-directed replication accuracy, difficulties abound, as the production of a non-specific enzyme would favor short free-riding replicators that benefit from the enzyme without encoding it [14].

The search for solutions to these puzzles has set the agenda for theoretical research on prebiotic evolution since Eigen’s seminal work on the evolution of self-replicating molecules [3]. The coexistence of replicators can be ensured by assuming a cyclic reaction scheme, called hypercycle, in which each replicator would help the next one to replicate, in a regulatory cycle closing on itself [4,15,16]. However, this scheme is not resistant to the presence of free-riders [17]. An alternative scheme temporarily confines the templates into packages or prebiotic vesicles that are considered viable provided they contain a set of distinct functional templates [17,18,19,20]. This scenario has been tested experimentally in in vitro molecular systems [21], which confirmed that transient compartmentalization is capable of maintaining functional replicators despite the presence of free-riders (see [22,23,24] for a theoretical analysis).

The population biology of replicators, which captures their interactions and dynamics and, most importantly, determines what types of mutant replicators can arise and evolve in a resident population, is essential to the study of the complexification of life. Here, we revisit a classic study on the population biology of early replicators [14] from an evolutionary game-theoretic perspective. In particular, we focus on the effects of demographic noise that arises when the population of replicators is finite. Following the usual population genetics approach to finite populations [25], we concentrate on the probability that one of the replicator types fixates and on the unconditional mean time to fixation. Crucially, we borrow from evolutionary game theory the stochastic algorithms that simulate the trajectories of finite populations and recover the replicator equations in the infinite population limit [26,27].

In all the competition scenarios studied, involving template-directed (non-enzymatic) replicators and protein-mediated (enzymatic) replicators, we find a threshold at which the replicators are neutral: away from the threshold, one of the replicator types dominates. Near the threshold (i.e., in the quasi-neutral regime), we use finite-size scaling [28] to obtain explicit expressions of the fixation probability and mean fixation time in terms of the model parameters. Since the emergence of enzyme-producing replicators is impossible in a well-mixed population of free-riding replicators, we consider a population structured in temporarily formed random groups of fixed size *n*. We argue that this problem is identical to the weak-altruism version of the *n*-player prisoner’s dilemma [29] and offer a thorough study of the model near the threshold, which in this case is given by the weak-altruism condition: the reward for altruistic behavior equals the cost of performing it [30].

Following the seminal works on the population dynamics of early replicators [3,14], in this study we consider only the competition between two different types of replicators. The reason for this is that in a well-mixed unstructured population, two different types of replicators cannot coexist [13], so the equilibrium scenario will always be a homogeneous population composed of a single type of replicator, and competition arises with the appearance of a mutant or migrant of a different type. The probability of two or more distinct mutants, or two or more migrants from different populations, resulting in a competitive scenario with more than two replicator types is negligible. This is also the reason why the non-invadability conditions of the resident population are so important to characterize the possible equilibrium scenarios [5].

The remaining sections are organized as follows. In Section 2, we study the competition between different types of replicators in well-mixed populations, which we call non-structured populations. Replicators are considered of different types not only because of their mode of replication (i.e., template-directed or protein-mediated), but also if they have the same mode of replication but different growth parameters. We present the results for both the replicator equation framework [31], which is valid for infinitely large populations, and the stochastic algorithms used to simulate finite populations. In Section 3, we consider the problem of the evolution of enzymatic replication in the worst-case scenario of a non-specific enzyme that can promote the replication of producers and free-riders with equal efficiency. This problem is considered in a structured population scenario where replicators are confined to small compartments or protocellular structures, so that a replicator can only benefit from the enzyme if there are enzyme producers in the same compartment. Our original contribution to the population dynamics of replicators in both non-structured and structured populations is the use of evolutionary game theory stochastic algorithms to study the dynamics for finite populations. In Section 4, we summarize our main findings and present some concluding observations.

## 2. Non-Structured Populations

We consider a well-mixed finite population of size *M* consisting of replicators of two different types. The population is well-mixed in the sense that each replicator can interact with every other replicator in the population. More importantly, in the case of enzymatic replicators, we assume that the enzymes benefit all replicators in the population that have an affinity for them.

### 2.1. Malthusian vs. Malthusian Replicators

A Malthusian or template-directed replicator is a replicator that follows Malthusian dynamics, i.e., in the absence of density regulation, its abundance grows exponentially with time, with growth rates determined by the balance between birth and death processes [14]. The competitive setup arises when we impose the constraint that the total number of replicators is kept at some constant number [3], so that the interaction between different types of replicators can be seen as a zero-sum game. In particular, this constraint allows us to completely describe the population in terms of the frequencies $x_{a}\in \left[0,1\right]$ with $\sum_{a}x_{a}=1$ of the different types of replicators. In the case of two Malthusian competitors with intrinsic growth rates $r_{a}$ and $r_{b}$, the dynamics in the limit M→∞ is governed by the replicator equations

(1) $$\begin{array}{ccc} \frac{dx_{a}}{dt} & = & \alpha x_{a}\left(r_{a}-\psi \right), \end{array}$$

(2) $$\begin{array}{ccc} \frac{dx_{b}}{dt} & = & \alpha x_{b}\left(r_{b}-\psi \right), \end{array}$$

where $\psi =r_{a}x_{a}+r_{b}x_{b}$ is the mean fitness of the population that ensures the constraint $x_{b}+x_{a}=1$ is satisfied for all *t*. Here, the parameter α determines the time scale and is important for connecting the deterministic formulation of the replicator equation with the stochastic dynamics, as we will see next. Writing $x_{b}$ in terms of $x_{a}$ yields

(3) $$\frac{dx_{a}}{dt}=\alpha \left(r_{a}-r_{b}\right)x_{a}\left(1-x_{a}\right),$$

from where we can immediately see that $x_{a}=0$ and $x_{a}=1$ are the only equilibrium solutions of the replicator dynamics. In particular, the fixed point $x_{a}=1$ is stable if $r_{a}>r_{b}$ and unstable if $r_{a}<r_{b}$, in which case the fixed point $x_{a}=0$ is stable. If $r_{a}=r_{b}$, the dynamics freezes at the initial condition, i.e., $x_{a}\left(t\right)=x_{a}\left(0\right)$ for all times. Thus, for an infinite population, the replicator type with the higher intrinsic growth rate wins the competition. This conclusion can also be reached by explicitly solving Equation (3),

(4) $$x_{a}\left(t\right)=\frac{x_{a}\left(0\right)}{x_{a}\left(0\right)+\left[1-x_{a}\left(0\right)\right]\exp\left[-\alpha (r_{a}-r_{b})t\right]}.$$

The deterministic dynamics involving the competition between two (or more) Malthusian replicators is well known [3,14], so our focus is on the finite population effects, i.e., the effects of demographic noise on the competition between replicators. The above results for the deterministic limit are essential to validate the finite population simulations, which should recover the deterministic results as the population size *M* increases. One difficulty here is that the Gillespie algorithm [32], which is the standard numerical method for simulating the stochastic time evolution of coupled chemical reactions, is not well suited for simulating systems with hard constraints, since to satisfy the constraint of a fixed total number of replicators, two different reactions must occur simultaneously. Here we borrow the stochastic dynamics used in evolutionary game theory, which recovers the replicator equation in the limit of infinite population size [26,27].

We note that the replicator equation is always nonlinear by construction due to the constant total density constraint, but it is possible to find explicit analytical solutions in many competition scenarios [31], as done above. There are also fractional versions of the replicator equation (see, e.g., [33,34]) for which even finding their numerical solutions is challenging [35]. Rather than solving the replicator equation numerically, we focus on stochastic simulations of finite populations of replicators, which recover the results of the (non-fractional) replicator equation for infinite population sizes.

The stochastic dynamics for the competition between two types of Malthusian replicators in a population of finite and fixed size *M* is as follows. Randomly select two different replicators *i* and *j*, with i,j=1,…,M. We will refer to replicator *i* as the challenged replicator and to replicator *j* as the challenger. The challenger replaces the challenged replicator with probability $r_{j}/\left(r_{j}+r_{i}\right)$, where $r_{i}$ and $r_{j}$ take values $r_{a}$ or $r_{b}$, depending on the type of replicators *i* and *j*. Regardless of whether the challenger succeeds in replacing the challenged replicator or not, the time *t* is increased by the time step δt. Then a new pair of replicators is selected and the process is repeated until all replicators in the population are either of type *a* or type *b*. In case of replacement, the challenger makes a copy of itself, which replaces the challenged replicator. In the Appendix A, we prove that this stochastic dynamics leads to the replicator Equation (3) in the limit M→∞ if we set δt=1/M and $\alpha =1/(r_{a}+r_{b})$.

Since only the ratio between the intrinsic growth parameters $r_{a}$ and $r_{b}$ appears in both the deterministic and the stochastic formulations, it is convenient to introduce the reduced variable

(5) $$\rho =\frac{r_{a}}{r_{a}+r_{b}},$$

which is restricted to the interval [0,1]. In terms of this variable, the replicator Equation (3) is rewritten as

(6) $$\frac{dx_{a}}{dt}=\left(2\rho -1\right)x_{a}\left(1-x_{a}\right).$$

**Figure 1:**
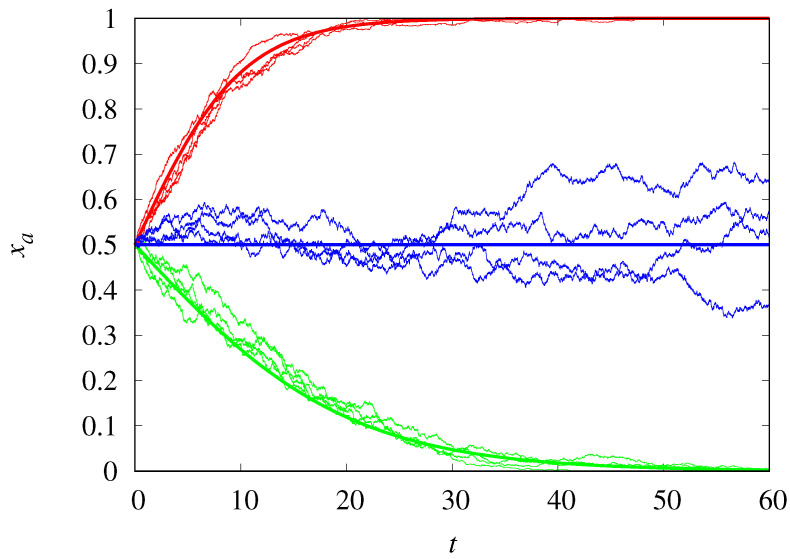
Frequency of Malthusian replicators of type *a* as a function of time for ρ=0.6 (red curves), ρ=0.5 (blue curves), and ρ=0.45 (green curves). The jagged thin curves are trajectories of the stochastic dynamics for M=1000, and the smooth thick curves are the deterministic results. The initial condition is $x_{a}\left(0\right)=0.5$.

Figure 1 compares the trajectories of the stochastic dynamics for populations of size M=1000 with the deterministic results. The agreement is excellent, as expected, except at the threshold $\rho_{c}=0.5$, since the stochastic trajectories will eventually reach one of the absorbing states $x_{a}=1$ or $x_{a}=0$, while the deterministic dynamics is frozen at the initial condition $x_{a}\left(0\right)=0.5$. For not too large *M*, the demographic noise can push the dynamics into the opposite absorbing state predicted by the deterministic equation. Moreover, even when starting from the same initial state, the noise can lead the stochastic trajectories to different absorbing states. Note that the closer ρ is to 0.5, the longer it takes to converge to the absorbing state.

**Figure 2:**
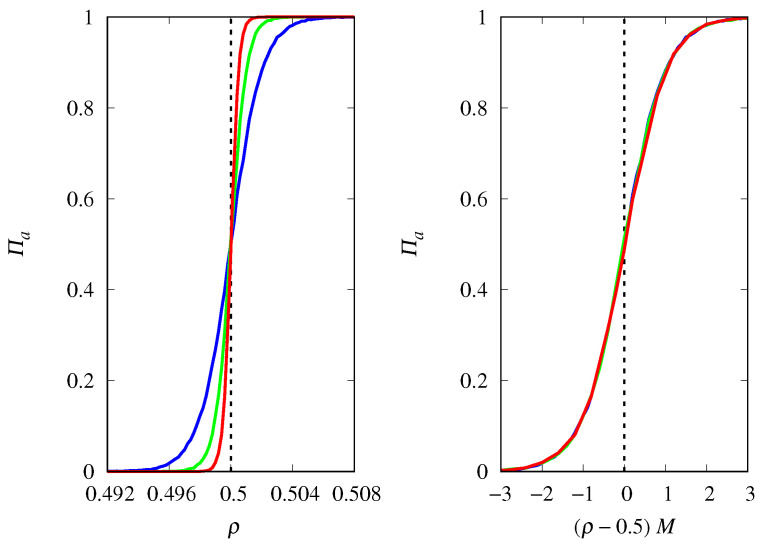
(**Left**) Probability of fixation $\Pi_{a}$ of Malthusian replicators of type *a* as a function of $\rho =r_{a}/\left(r_{a}+r_{b}\right)$ for M=2000 (red curve), M=1000 (green curve), and M=500 (blue curve). The vertical dashed line indicates the threshold $\rho_{c}=0.5$ beyond which the fixed point $x_{a}=1$ is stable for M→∞. (**Right**) $\Pi_{a}$ as a function of the scaled variable $(\rho -\rho_{c})M$. The initial condition is $x_{a}\left(0\right)=0.5$.

To quantify the effect of demographic noise, we consider the probability of fixation of replicators of type *a*, denoted by $\Pi_{a}$, which is estimated as the fraction of $10^{4}$ independent trajectories of the stochastic dynamics that are attracted to the absorbing state consisting only of replicators of type *a*. The results in Figure 2 show that the magnitude of the effect of demographic noise depends on the population size *M* and on the proximity of ρ to the threshold $\rho_{c}=0.5$. In fact, the scaling assumption $\Pi_{a}\approx f\left[(\rho -\rho_{c})M\right]$ perfectly describes this dependence in the threshold region for large *M*, as shown by the collapse of the curves for different *M* when ρ is properly shifted and scaled [28]. For other applications of the curve collapse method, see [36,37]. Here, f(u) is a scaling function such that f(u)→1 when u→∞ and f(u)→0 when u→−∞ (see Equation (7) for the explicit form of this scaling function). The steepness of the threshold transition for functions such as those shown in Figure 2 is estimated by their derivatives at the threshold. Recall that these derivatives are the slopes of the tangent line to the graph of $\Pi_{a}$ versus ρ at the threshold: a large derivative indicates a sharp threshold transition and a small derivative indicates a smooth threshold transition. Since $\Pi_{a}^{\prime }\left(\rho_{c}\right)\approx Mf^{\prime }\left(0\right)$, we conclude that the steepness of the threshold transition increases linearly with the population size *M*.

**Figure 3:**
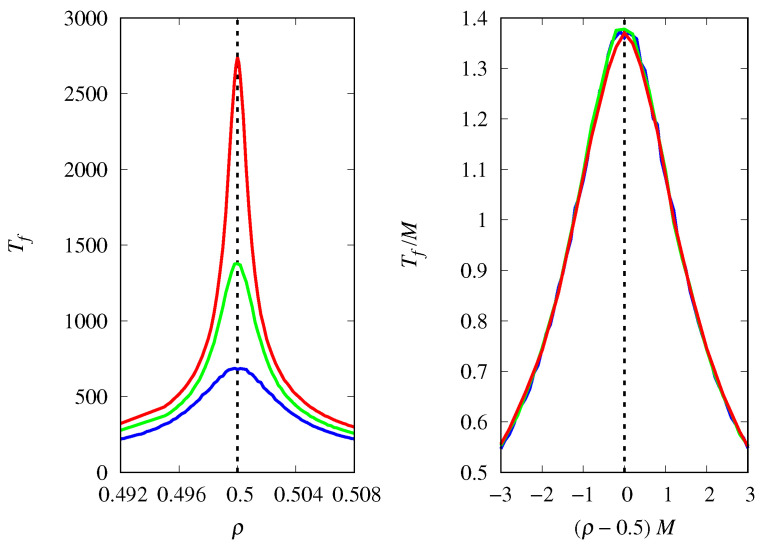
(**Left**) Mean time for fixation $T_{f}$ of either type of Malthusian replicators as a function of $\rho =r_{a}/\left(r_{a}+r_{b}\right)$ for M=2000 (red curve), M=1000 (green curve), and M=500 (blue curve). The vertical dashed line indicates the threshold $\rho_{c}=0.5$. (**Right**) Scaled mean fixation time $T_{f}/M$ as a function of the scaled variable $(\rho -\rho_{c})M$. The initial condition is $x_{a}\left(0\right)=0.5$.

Another important quantity to characterize the stochastic dynamics is the unconditional mean fixation time $T_{f}$, i.e., the mean time for the dynamics to reach any absorbing state, which is shown in Figure 3. The results indicate that $T_{f}$ diverges linearly with *M* at the threshold $\rho_{c}$ in the limit M→∞. Away from the threshold region, we find that $T_{f}$ diverges with lnM in this limit.

**Figure 4:**
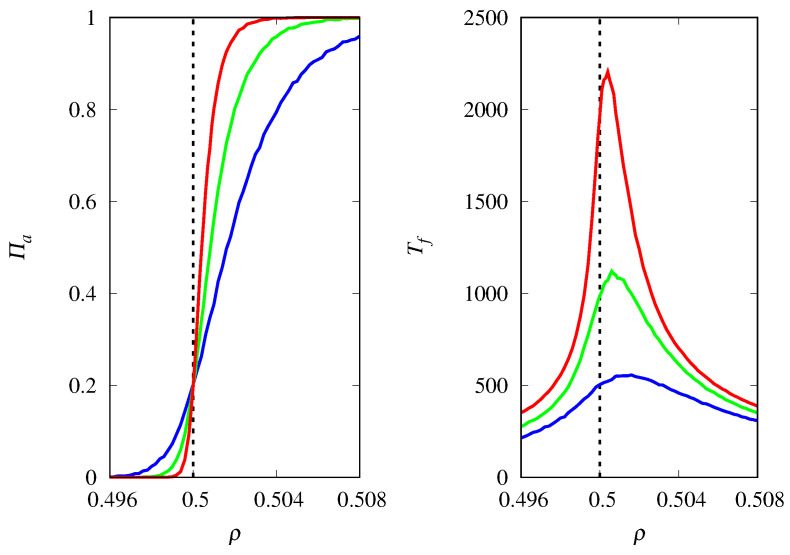
(**Left**) Probability of fixation $\Pi_{a}$ of Malthusian replicators of type *a* as a function of $\rho =r_{a}/\left(r_{a}+r_{b}\right)$ for M=2000 (red curve), M=1000 (green curve), and M=500 (blue curve). (**Right**) Mean time for fixation $T_{f}$ of either type of replicators as a function of ρ. The vertical dashed lines indicate the threshold $\rho_{c}=0.5$. The initial condition is $x_{a}\left(0\right)=0.2$.

The reason why the fixation probability is invariant to the change ρ→1−ρ and $\Pi_{a}\to 1-\Pi_{a}$, and $T_{f}$ is symmetric around the threshold $\rho_{c}$, is that $x_{a}\left(0\right)=0.5$ in Figure 2 and Figure 3, i.e., at t=0 the replicators are assigned to types *a* or *b* with equal probability. Figure 4 shows the results for $x_{a}\left(0\right)=0.2$. Recall that for the competition between two Malthusian replicators, the threshold occurs at $\rho_{c}=0.5$, regardless of the initial condition $x_{a}\left(0\right)$. The symmetry about the vertical line at $\rho =\rho_{c}$ is lost, since for finite *M* and fixed ρ, replicators of type *a* are less likely to be fixated due to their initial disadvantage. In addition, replicators of type *a* take longer to reach fixation than replicators of type *b*, as expected. Note that for finite *M*, the maximum of $T_{f}$ does not occur at $\rho =\rho_{c}$, but it moves in the direction of the threshold $\rho_{c}$ as *M* increases. The dependence of $\Pi_{a}$ and $T_{f}$ on *M* in the vicinity of the threshold is the same as that discussed above for $x_{a}\left(0\right)=0.5$.

Note that at the threshold, or equivalently for $r_{a}=r_{b}$, the curves for different *M* intersect at $\Pi_{a}=x_{a}\left(0\right)$ (see the left panels of Figure 2 and Figure 4), which gives the fixation probability of replicators of type *a* in the limit M→∞. Thus, the scaling function at the threshold is $f\left(0\right)=x_{a}\left(0\right)$. This is the classical result for the probability of fixation of a neutral mutant [25]. Even better, if we set the selective advantage *s* of type *a* replicators to s=2ρ−1, then Kimura’s probability of fixation [25]

(7) $$\begin{array}{ccc} \Pi_{a} & = & \frac{1-\exp\left[-2Msx_{a}\left(0\right)\right]}{1-\exp\left[-2Ms\right]}\\ & = & \frac{1-\exp\left[-4M\left(\rho -1/2\right)x_{a}\left(0\right)\right]}{1-\exp\left[-4M\left(\rho -1/2\right)\right]} \end{array}$$

fits the simulation results perfectly. It is interesting to note that this equation is valid in the limit of large *M* and small *s*, which are exactly the conditions used in our finite-size scaling analysis. The connection between the stochastic dynamics for the competition between Malthusian replicators and Kimura’s diffusion equation approach to population genetics, which led to Equation (7), can be made explicit by considering the 1/M corrections in the analytical treatment of the stochastic dynamics [26]. Kimura’s diffusion theory predicts that the mean fixation time of an allele with a small selective advantage scales with lnM, but for neutral alleles (i.e., s=0 or ρ=1/2), $T_{f}$ scales with *M* [25], which is consistent with the results in Figure 3.

### 2.2. Hypercyclic vs. Hypercyclic Replicators

Hypercyclic or enzymatic replicators follow a nonlinear growth equation, even without the constant density constraint, due to the presence of a protein catalyst (enzyme) that promotes their replication [4]. The nonlinearity occurs because the catalysts are produced by the hypercyclic replicators themselves. These replicators exhibit characteristics that differ from Malthusian replicators, such as explosive growth and the potential for “once-forever” decisions, where once a replicator type becomes fixed in a population, it cannot be replaced by another more efficient hypercyclic replicator.

In the deterministic regime, a simplified scenario for the competition between hypercyclic replicators of types *a* and *b* is described by the replicator equations [14]

(8) $$\begin{array}{ccc} \frac{dx_{a}}{dt} & = & \alpha x_{a}\left(c_{a}x_{a}-\psi \right), \end{array}$$

(9) $$\begin{array}{ccc} \frac{dx_{b}}{dt} & = & \alpha x_{b}\left(c_{b}x_{b}-\psi \right), \end{array}$$

where $x_{a}$ and $x_{b}$ are the frequencies of the two replicator types in an infinite population. Here, $c_{a}$ and $c_{b}$ represent the beneficial effect of protein-mediated replication. In addition, these parameters include the production of specific enzymes from each replicator type. As before, $\psi =c_{a}x_{a}^{2}+c_{b}x_{b}^{2}$ guarantees that the constraint $x_{a}+x_{b}=1$ is met for all times, and α is the time scale. Eliminating $x_{b}$ we get

(10) $$\frac{dx_{a}}{dt}=\alpha \left(c_{a}+c_{b}\right)x_{a}\left(1-x_{a}\right)\left(x_{a}-1+\chi \right),$$

with

(11) $$\chi =\frac{c_{a}}{c_{a}+c_{b}}.$$

This equation has three fixed points: the fixed points $x_{a}=0$ and $x_{a}=1$ are always stable, while the unstable fixed point $x_{a}=1-\chi$ gives the boundary of the domains of attraction of the two stable fixed points. Therefore, a resident population of hypercyclic replicators of type *a* cannot be invaded by rare invaders of type *b*, even if $c_{b}\gg c_{a}$. In this sense, the fixation of a hypercyclic replicator in a population is a “once-forever” decision [3,4].

Explicit integration of Equation (10) yields (see Appendix B).

(12) $$\alpha \left(c_{a}+c_{b}\right)t=\frac{1}{\chi (1-\chi )}\ln\left[\frac{x_{a}-1+\chi }{x_{a}\left(0\right)-1+\chi }\right]-\frac{1}{1-\chi }\ln\left[\frac{x_{a}}{x_{a}\left(0\right)}\right]-\frac{1}{\chi }\ln\left[\frac{1-x_{a}}{1-x_{a}\left(0\right)}\right],$$

which allows us to plot $x_{a}$ as a function of *t* without solving Equation (10) numerically. Note that since $x_{a}\in \left[0,1\right]$ in the competitive scenario, Equation (12) does not exhibit explosive growth (i.e., divergence at finite *t*). For fixed $x_{a}\left(0\right)$, the transition between the different equilibrium regimes occurs at the threshold $\chi_{c}=1-x_{a}\left(0\right)$.

As before, the results for the deterministic regime are well known, and we have presented them here because they are necessary for validating the finite population simulations that are the focus of this paper. The stochastic dynamics that reproduces the replicator Equation (10) in the infinite population limit is as follows. First, we randomly select the challenged replicator *i* and compute its instantaneous payoff $f_{i}$. This is done by randomly selecting another replicator and checking if it is of the same type as the challenged replicator. If so, we set $f_{i}=c_{a}$ if replicator *i* is of type *a*, and $f_{i}=c_{b}$ if replicator *i* is of type *b*. If not, we set $f_{i}=0$. (Note that the instantaneous payoff is determined by a coordination game [5], where a player gets a higher payoff by choosing the same action as its opponent.) Then we select the challenger replicator j≠i and compute its instantaneous payoff $f_{j}$ in the same way. The challenger replaces the challenged replicator with probability

(13) $$\frac{f_{j}-f_{i}}{\max(c_{a},c_{b})}$$

if $f_{j}>f_{i}$, otherwise the challenged replicator keeps its type. The denominator in this equation is chosen to ensure that the probability of replacement is less than or equal to 1. Time is updated using the time step δt=1/M, and another pair of challenged-challenger replicators is selected. The process is repeated until the population becomes homogeneous. In the limit M→∞, this stochastic dynamics is described by the replicator Equation (10) if we set the time scale as $\alpha =1/\max(c_{a},c_{b})$ [27]. With this setting, this equation depends only on the variable χ.

**Figure 5:**
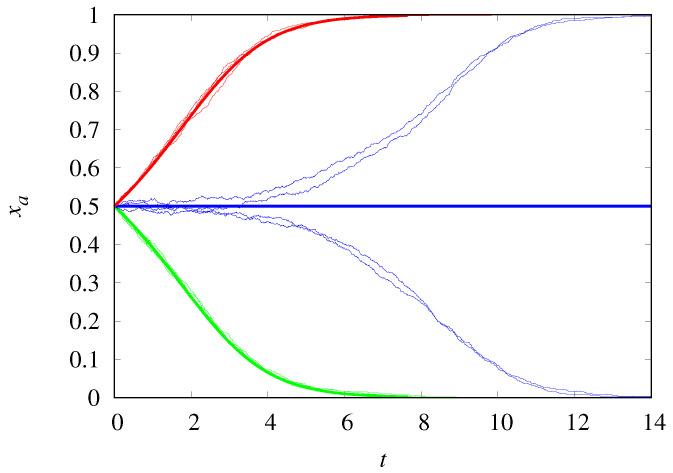
Frequency of hypercyclic replicators of type *a* as a function of time for χ=0.8 (red curves), χ=0.5 (blue curves), and χ=0.2 (green curves). The jagged thin curves are trajectories of the stochastic dynamics for M=1000, and the smooth thick curves are the deterministic results. The initial condition is $x_{a}\left(0\right)=0.5$.

Figure 5 shows the excellent agreement between the stochastic and deterministic trajectories when χ is far from the threshold $\chi_{c}=1-x_{a}\left(0\right)$. A comparison with Figure 1, which shows the time evolution of two competing Malthusian replicators, indicates that the dynamics reach equilibrium much faster for competing hypercyclic replicators, even at the threshold. This implies that for fixed *M* the effect of demographic noise leading to the fixation of one of the replicator types is more pronounced for hypercyclic replicators.

**Figure 6:**
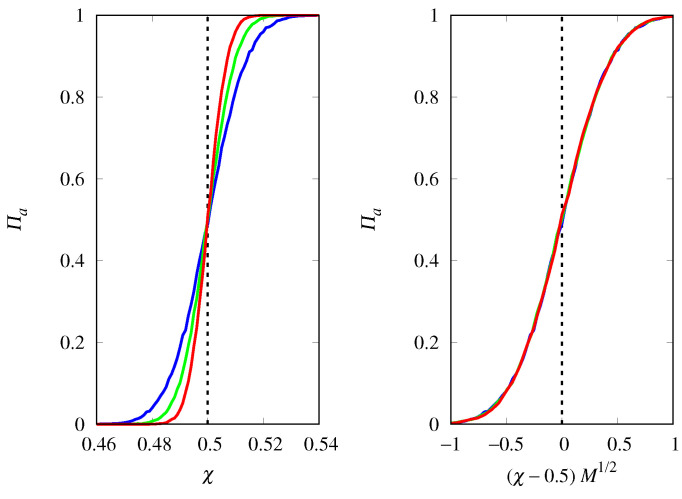
(**Left**) Probability of fixation $\Pi_{a}$ of hypercyclic replicators of type *a* as a function of $\chi =c_{a}/\left(c_{a}+c_{b}\right)$ for M=4000 (red curve), M=2000 (green curve), and M=1000 (blue curve). The vertical dashed line indicates the threshold $\chi_{c}=1-x_{a}\left(0\right)=0.5$. (**Right**) $\Pi_{a}$ as a function of the scaled variable $\left(\chi -\chi_{c}\right)M^{1/2}$.

In fact, Figure 6 shows that the fixation probability of the hypercyclic replicators of type *a* is given by the scaling relation $\Pi_{a}\approx g\left[\left(\chi -\chi_{c}\right)M^{1/2}\right]$ near the threshold and for large *M*, where g(u) is a scaling function such that g(u)→1 when u→∞ and g(u)→0 when u→−∞. This means that the steepness of the threshold transition increases as $M^{1/2}$ as *M* increases, indicating that much larger populations are needed to suppress demographic noise for hypercyclic replicators compared to Malthusian replicators. Another difference from the previous analysis is that, by varying the initial condition $x_{a}\left(0\right)$, we find $\Pi_{a}=1/2$ at the threshold $\chi_{c}=1-x_{a}\left(0\right)$. Therefore, the scaling function must be such that g(0)=1/2.

**Figure 7:**
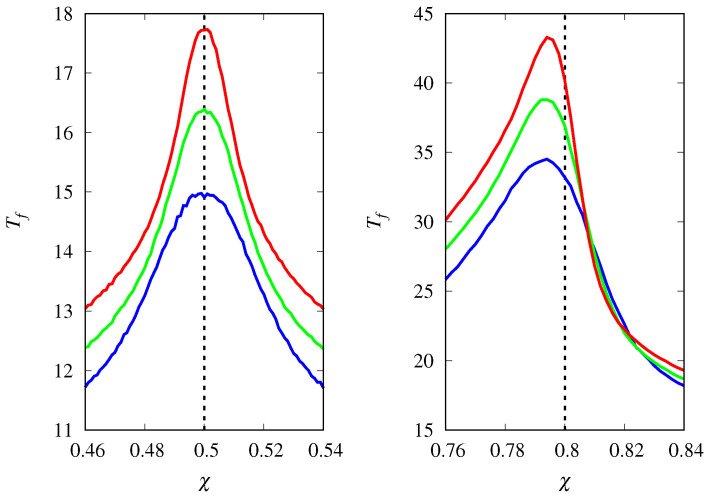
Mean time for fixation $T_{f}$ of either type of hypercyclic replicators as a function of $\chi =c_{a}/\left(c_{a}+c_{b}\right)$ for M=4000 (red curve), M=2000 (green curve), and M=1000 (blue curve). The vertical dashed line indicates the threshold $\chi_{c}=1-x_{a}\left(0\right)$. (**Left**) $x_{a}\left(0\right)=0.5$. (**Right**) $x_{a}\left(0\right)=0.2$.

**Figure 8:**
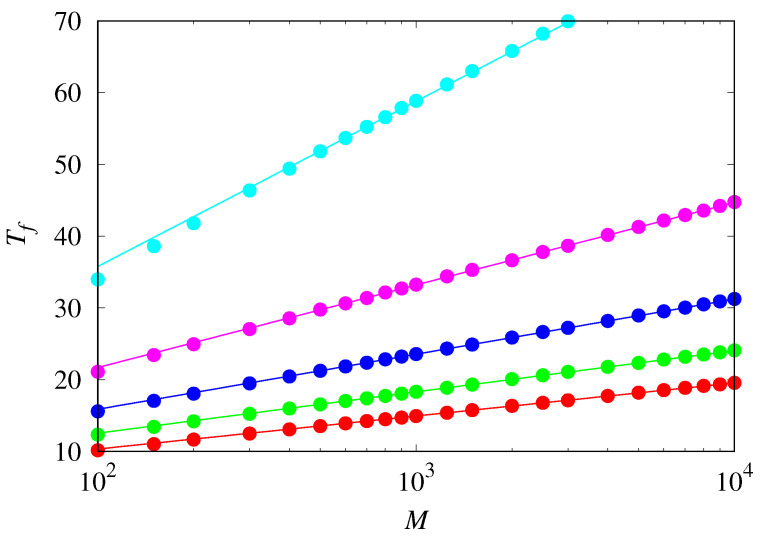
Mean fixation time $T_{f}$ of either type of hypercyclic replicators at the threshold $\chi_{c}=1-x_{a}\left(0\right)$ as a function of the population size *M* for (from bottom to top) $x_{a}\left(0\right)=0.5,0.6,0.7,0.8$, and 0.9. The lines are the fit $T_{f}=a_{\chi }+\ln M^{1/(1-\chi )}$ where $a_{\chi }$ is a fit parameter.

Figure 7 shows the mean fixation time for the competition between hypercyclic replicators near the threshold for two initial conditions. As hinted at in Figure 5, the dynamics reach the absorbing states very quickly, perhaps a reminiscence of the explosive growth characteristic of unrestrained hypercyclic replicators [3,4]. Note that doubling the value of *M* only increases $T_{f}$ by an amount of about 2. In fact, Figure 8 shows that $T_{f}$ increases with the logarithm of *M*, viz., $T_{f}∼\frac{1}{1-\chi }\ln M$ for large *M*.

### 2.3. Hypercyclic vs. Malthusian Replicators

We consider hypercyclic replicators to have frequency $x_{a}$ and protein-mediated growth rate $c_{a}$, while Malthusian replicators have frequency $x_{b}$ and intrinsic growth rate $r_{b}$. Thus, here the subscript *a* refers to hypercyclic replicators and the subscript *b* to Malthusian replicators. It is interesting to find out how $\Pi_{a}$ and $T_{f}$ scale with *M* in this case, given the stark differences in scaling in the two previous competition scenarios. In the deterministic limit, the competition between these two types of replicators is described by the replicator equations [14]

(14) $$\begin{array}{ccc} \frac{dx_{a}}{dt} & = & \alpha x_{a}\left(c_{a}x_{a}-\psi \right), \end{array}$$

(15) $$\begin{array}{ccc} \frac{dx_{b}}{dt} & = & \alpha x_{b}\left(r_{b}-\psi \right), \end{array}$$

where $\psi =c_{a}x_{a}^{2}+r_{b}x_{b}$ ensures that $x_{a}+x_{b}=1$ for all times, and α is the time scale as before. Eliminating $x_{b}$, we get

(16) $$\frac{dx_{a}}{dt}=\alpha c_{a}x_{a}\left(1-x_{a}\right)\left(x_{a}-\eta \right),$$

with

(17) $$\eta =\frac{r_{b}}{c_{a}}.$$

The fixed points $x_{a}=0$ and $x_{a}=1$ are stable provided that η<1, in which case the unstable fixed point $x_{a}=\eta$ separates the domains of attraction of the stable fixed points. This is the same bistability scenario found in the competition between two hypercyclic replicators. If η>1, the only stable fixed point is $x_{a}=0$, which means that rare Malthusian replicators can invade a resident population of hypercyclic replicators. In this sense, the fixation of hypercyclic replicators is not a “once-forever” decision. However, the idea of introducing protein-mediated replication is that it is much more efficient than direct template replication, so we should have $c_{a}\gg r_{b}$ or η≪1, instead.

The stochastic dynamics in this case is as follows. First, we randomly select the challenged replicator *i*. The instantaneous payoff $f_{i}$ depends on the nature of the replicator *i*. If it is a hypercyclic replicator, we randomly choose another replicator and check its nature: if it is also a hypercyclic replicator, we set $f_{i}=c_{a}$, and if it is a Malthusian replicator, we set $f_{i}=0$. If the challenged replicator *i* is a Malthusian replicator we set $f_{i}=r_{b}$. Then we select the challenger replicator j≠i and compute its instantaneous payoff $f_{j}$ in the same way. As before, the challenger replaces the challenged replicator with probability

(18) $$\frac{f_{j}-f_{i}}{\max(c_{a},r_{b})}$$

if $f_{j}>f_{i}$, otherwise the challenged replicator maintains its type. Time is updated as before, and the process is repeated until the population becomes homogeneous. If we set the time scale to $\alpha =1/\max(c_{a},r_{b})$, this stochastic dynamics leads to the replicator Equation (16) in the limit M→∞ [27].

**Figure 9:**
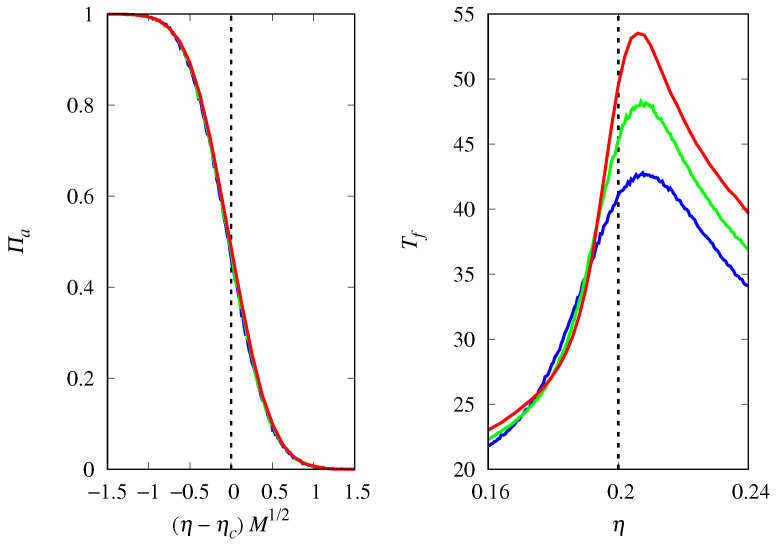
(**Left**) Probability of fixation $\Pi_{a}$ of hypercyclic replicators competing against Malthusian replicators as a function of the scaled variable $\left(\eta -\eta_{c}\right)M^{1/2}$, where $\eta =r_{b}/c_{a}$ and $\eta_{c}=x_{a}\left(0\right)=0.2$, for M=4000 (red curve), M=2000 (green curve), and M=1000 (blue curve). (**Right**) Mean fixation time $T_{f}$ as a function of η. The vertical dashed line indicates the threshold $\eta_{c}$.

Figure 9 shows that the competition between hypercyclic and Malthusian replicators is qualitatively similar to the competition between two hypercyclic replicators. In particular, the steepness of the transition at the threshold $\eta_{c}=x_{a}\left(0\right)$ increases as $M^{1/2}$, and the fixation time increases as lnM as *M* increases. Thus, the hypercyclic replicator determines the strength of the demographic noise.

The important lesson from the competition between hypercyclic and Malthusian replicators is that a few hypercyclic replicators cannot invade a resident population of Malthusian replicators. In addition, replicators that do not produce the enzyme are likely to benefit from it as well. Since Malthusian (non-enzymatic, template-directed) replication is likely to have arisen first in the evolution of life, these are major hurdles to the evolution of more efficient enzymatic replication. In the following, we discuss how compartmentalization of the replicators can address these problems.

## 3. Structured Populations

A possible solution to the evolution of enzymatic replication is to assume that the replicators are temporarily confined in groups, e.g., rock crevices, suspended clay particles, or suspended water droplets [38], so that the enzyme producers experience the benefits of the enzyme more strongly [14]. Thus, group confinement produces the positive assortment among enzyme producers (cooperators) necessary for their maintenance [39]. Another way to produce this positive assortment is through the spatial localization of replicators, since the aggregation of cooperators into clusters may protect those in the bulk from exploitation by free-riders, i.e., replicators that benefit from the enzyme but do not encode it (see, e.g., [40,41,42]). Here, we will consider the positive assortment resulting from temporarily formed random groups, which has a long tradition in theoretical prebiotic evolution studies [14,17,18,23,43]. In fact, this approach combines the first studies of the origin of life, which focused on the emergence of protocellular structures (e.g., Oparin’s coacervates [44]), with the more modern approach, which focuses on the replication process [3], a key component of any system, living or not, that evolves under natural selection. In this sense, we say that the population is structured, i.e., the replicators are confined in protocellular structures, which we call groups.

In particular, we use Wilson’s trait group formulation [45] to model the dynamics of compartmentalized replicators. In this formulation, the fitness of the replicators are determined locally within their groups of fixed size *n*, but there is no intragroup competition and the mean fitness of the group plays no role in the evolutionary process. Competition takes place in the population at large, with individuals from all groups randomly selected to form the next generation with probability proportional to their fitness, i.e., competition happens when all groups merge into a common pool of replicators [45]. This contrasts with a more recent model of transient compartmentalization [22,23,24], which includes a maturation phase with intragroup competition that leads to the disappearance of cooperators from any group containing free-riders: only groups formed only by cooperators can maintain cooperation. In addition, the size of each group grows at a rate given by the group fitness, so that the all-cooperators groups eventually contribute more offspring to the common pool. As in Wilson’s formulation, groups are formed by randomly selecting replicators from a common pool. A remarkable aspect of Wilson’s formulation is that it is closely related to the evolutionary game theory approach to *n*-player public goods games [46], as will become clear when we formulate the enzyme production problem as an *n*-player evolutionary game.

### 3.1. Enzyme-Production as a Public Goods Game

As in the previous analyses, we consider a scenario with two types of replicators. Type *a* replicators, which produce an enzyme that increases the replication rate of all replicators (including themselves) in the group, but at the cost of decreasing their template-directed replication rates, and type *b* replicators (free-riders), which do not produce the enzyme but benefit from it. This differs from the hypercyclic vs. Malthusian replicator scenario discussed earlier, as the Malthusian replicators do not benefit from the enzyme, i.e., the enzyme is specific to hypercyclic replicators. Although this is a best-case scenario for the emergence of enzyme-producing replicators, these replicators cannot invade a resident population of Malthusian replicators in an unstructured population, as shown before. Since the amount of enzyme is proportional to the number of producers, the instantaneous payoff of a replicator of type *a* in a group of size *n* that contains k+1 replicators of type *a* is [14]

(19) $$f_{a}=r_{a}+c_{a}\frac{k+1}{n},$$

with k=0,…,n−1. The instantaneous payoff of a replicator of type *b* in a group with *k* replicators of type *a* is [14]

(20) $$f_{b}=r_{b}+c_{b}\frac{k}{n},$$

with k=0,…,n−1, since at least one member of the group must be a replicator of type *b*. We have $r_{a}<r_{b}$ to account for the cost of producing the enzyme. The parameters $c_{a}$ and $c_{b}$ represent the beneficial effect of enzyme-mediated replication. In particular, $c_{b}=0$ implies that the enzyme is specific to the replicator that produced it, as in the competition between hypercyclic and Malthusian replicators discussed earlier. However, it seems more plausible to assume that the ancestral enzymes were some kind of general catalysts that would facilitate the replication of a wide range of replicators, so in the following we will assume the worst-case scenario of a non-specific enzyme and set $c_{a}=c_{b}$. Thus, the enzyme is considered a public good that is shared equally among the members of the group. Note that the instantaneous payoffs (19) and (20) are the growth rates of replicators of type *a* and *b*, respectively, having a term proportional to the concentration of enzymes in the group, which in turn is proportional to the concentration of enzyme-producing replicators in the group [14].

With this parameterization, the problem reduces to the *n*-player prisoner’s dilemma [29,47]. In the terminology of this game, if we set the baseline payoff to $r_{b}$, then the cooperator (i.e., the type *a* replicator) contributes an amount $r_{b}-r_{a}$ to the public goods, which is then multiplied by a factor $c_{a}/\left(r_{b}-r_{a}\right)>1$, and the resulting amount $c_{a}$ is divided among the *n* players. The free-rider (i.e., the type *b* replicator) contributes nothing ($r_{b}-r_{b}=0$) to the public goods, but gets its share of these goods.

Note that the payoff of type *b* replicators is always greater than the payoff of type *a* replicators in the same group, but when comparing the payoffs of replicators in different groups, it is possible for type *a* replicators to get an advantage over type *b* replicators. For this reason, cooperation can develop in the temporary group scenario, where competition takes place in the population at large [45]. However, if groups are formed randomly from a pool of dispersers (i.e., there is no positive assortment among replicators of type *a*), then cooperation can progress only in the so-called weak altruism scenario, where the return to altruistic behavior ($c_{a}/n$) exceeds the cost ($r_{b}-r_{a}$) of performing it [30]. For the payoffs (19) and (20), this condition corresponds to

(21) $$\frac{c_{a}}{n}>r_{b}-r_{a}.$$

This inequality will be explicitly derived in Section 3.3 as a condition for the stability of the all-cooperators equilibrium solution of the replicator equation. However, in the strong altruism scenario, where a cooperator does not benefit from its contribution to public goods, the evolution of cooperation requires positive assortment among cooperators [39,48], punishment of free-riders [49], or biparental sexual reproduction [50].

### 3.2. Stochastic Dynamics

The relation between the imitation dynamics and Wilson’s trait group formulation [45] is better appreciated for finite populations. As done before, the first step is to randomly select the challenged replicator *i*. To compute its instantaneous payoff $f_{i}$, we first need to create its play group, so we randomly select other n−1 replicators in the population without replacement. Next, we just have to determine the type of the challenged replicator and count the number of replicators of type *a* in its play group: its instantaneous payoff $f_{i}$ is given by Equation (19) or (20), depending on its type. Then we randomly select the challenger replicator j≠i and calculate its instantaneous payoff $f_{j}$ following the same procedure. Since n≪M, it is unlikely that challenger and challenged replicators will be in the same play group, but this is inconsequential. The probability that the challenger replicator replaces the challenged replicator is

(22) $$\frac{f_{j}-f_{i}}{\Delta f_{\max}},$$

if $f_{j}>f_{i}$, and 0 otherwise. Here, $\Delta f_{\max}$ is chosen so as to guarantee that this probability is not greater than 1. To compute this quantity, we need to know what are the group configurations that maximize and minimize a replicator instantaneous payoff. These configurations depend on whether the weak altruism condition (21) is satisfied or not. If this condition is satisfied, then the maximum individual payoff is $r_{a}+c_{a}$, obtained by a replicator of type *a* in a group with n−1 other replicators of type *a*, and the minimum individual payoff is $r_{b}$, obtained by a replicator of type *b* in a group with n−1 other replicators of type *b*. So $\Delta f_{\max}=c_{a}-\left(r_{b}-r_{a}\right)$. If condition (21) is violated, then the maximum individual payoff is $r_{b}+c_{a}\left(n-1\right)/n$ obtained by a replicator of type *b* in a group with n−1 replicators of type *a*, and the minimum individual payoff is $r_{a}+c_{a}/n$ obtained by a replicator of type *a* in a group with n−1 replicators of type *b*. So $\Delta f_{\max}=r_{b}-r_{a}+c_{a}\left(n-2\right)/n$. Once the replicator *i* is probed, time is updated with the time step δt=1/M and the whole procedure is repeated until fixation occurs.

Thus, the imitation dynamics exhibits the two main features of Wilson’s trait group formulation: the two competing replicators are randomly selected in the population at large, so there is no intragroup competition, and their fitness are obtained by playing a single round of the *n*-player prisoner’s dilemma in different play groups.

### 3.3. Deterministic Limit

In the deterministic regime, we assume an infinite population consisting of both types of replicators with frequencies $x_{a}$ and $x_{b}=1-x_{a}$. Consider a particular replicator of type *a*. Its payoff depends on the types of the n−1 other members of its group. Since groups are formed by randomly sampling from the population at large, the probability that there are exactly k=0,1,…,n−1 other replicators of type *a* in its group is given by the binomial distribution

(23) n−1kxakxbn−1−k.

Thus, the expected payoff $\pi_{a}$ of a replicator of type *a* is given by adding its payoff for all possible choices of the other members of its group, properly weighted by the probability of each choice, resulting in

(24) πa=∑k=0n−1n−1kxakxbn−1−kra+cak+1n=ra+caxa+(1−xa)can.

The expected payoff $\pi_{b}$ of a replicator of type *b* is obtained in the same way and is given by

(25) πb=∑k=0n−1n−1kxakxbn−1−krb+cakn=rb+caxa−xacan.

The replicator equations that govern the time evolution of the frequencies $x_{a}$ and $x_{b}$ are [31]

(26) $$\begin{array}{ccc} \frac{dx_{a}}{dt} & = & \alpha x_{a}\left(\pi_{a}-\psi \right), \end{array}$$

(27) $$\begin{array}{ccc} \frac{dx_{b}}{dt} & = & \alpha x_{b}\left(\pi_{b}-\psi \right), \end{array}$$

where α is the time scale and $\psi =x_{a}\pi_{a}+x_{b}\pi_{b}$ is the population mean fitness. Eliminating $x_{b}$, we obtain

(28) $$\frac{dx_{a}}{dt}=\alpha \left[\frac{c_{a}}{n}-\left(r_{b}-r_{a}\right)\right]x_{a}\left(1-x_{a}\right),$$

from which we can see that the fixed point $x_{a}=1$ is stable only if the weak altruism condition (21) is satisfied. The limit of infinitely large groups n→∞ describes the situation of a non-structured population, for which this condition is always violated and so $x_{a}=0$ is the only stable fixed point, as expected. We can easily write the explicit solution of Equation (28) as done for Equation (3), viz.,

(29) $$x_{a}\left(t\right)=\frac{x_{a}\left(0\right)}{x_{a}\left(0\right)+\left[1-x_{a}\left(0\right)\right]\exp\left[-\alpha \left(c_{a}/n-\left(r_{b}-r_{a}\right)\right)t\right]}.$$

In the limit M→∞, the stochastic dynamics recovers the replicator Equation (28) if we set the time scale to $\alpha =1/\Delta f_{\max}$ [27]. With this time scale, both the deterministic and the stochastic dynamics depend only on the reduced variable

(30) $$\gamma =\frac{c_{a}}{r_{b}-r_{a}},$$

so the threshold is $\gamma_{c}=n$ in the deterministic limit. At the threshold, we have $\pi_{a}=\pi_{b}$, so the two types of replicators are on equal footing in the population at large. In the terminology of the *n*-person prisoner’s dilemma, the parameter γ is the amplification factor of the cooperator’s contribution to the public goods [47].

### 3.4. Finite Population Simulations

**Figure 10:**
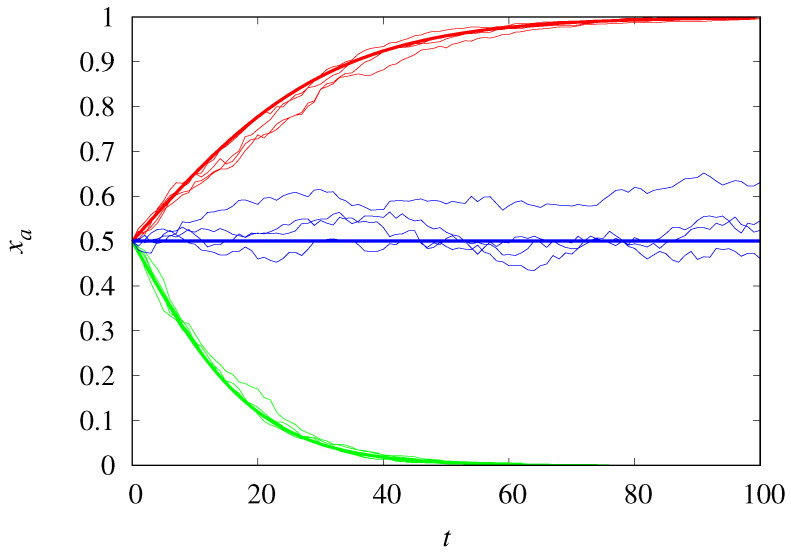
Frequency of replicators of type *a* (cooperators) as a function of time for groups of size n=4 and γ=5 (red curves), γ=4 (blue curves), and γ=3 (green curves). The jagged thin curves are trajectories of the stochastic dynamics for M=1000, and the smooth thick curves are the deterministic trajectories. The initial condition is $x_{a}\left(0\right)=0.5$.

Figure 10 shows the excellent agreement between the stochastic and deterministic trajectories for parameters far from the threshold $\gamma_{c}=n$, as expected. Even for balanced initial frequencies of the two replicator types, i.e., $x_{a}\left(0\right)=0.5$, the results indicate that the fixation of type *a* replicators takes slightly longer than the fixation of type *b* replicators.

**Figure 11:**
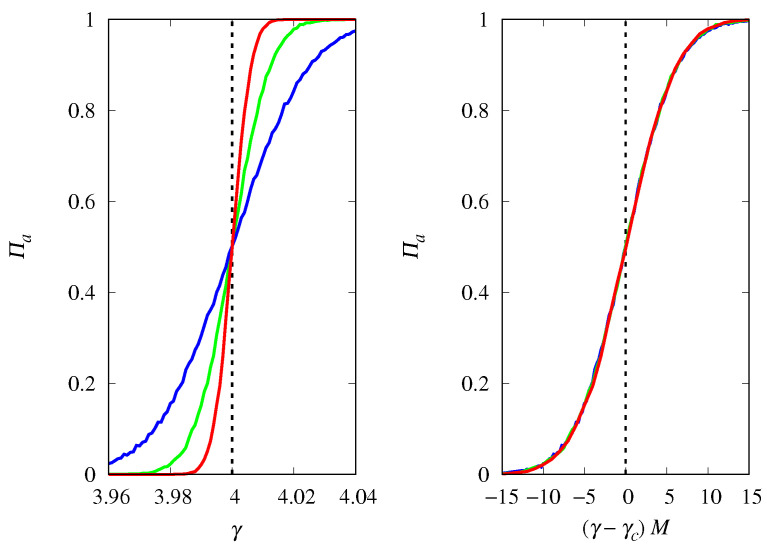
(**Left**) Probability of fixation $\Pi_{a}$ of replicators of type *a* (cooperators) as a function of γ for groups of size n=4 and M=1000 (red curve), M=500 (green curve), and M=250 (blue curve). The vertical dashed line indicates the threshold $\gamma_{c}=n$ beyond which the fixed point $x_{a}=1$ is stable for M→∞. (**Right**) $\Pi_{a}$ as a function of the scaled variable $(\gamma -\gamma_{c})M$. The initial condition is $x_{a}\left(0\right)=0.5$.

Figure 11 shows that the fixation probability of type *a* replicators is well approximated by $\Pi_{a}\approx h_{n}\left[(\gamma -\gamma_{c})M\right]$ near the threshold for large *M*, where $h_{n}\left(u\right)$ is a scaling function. Considering different initial conditions gives $h_{n}\left(0\right)=x_{a}\left(0\right)$, i.e., at the threshold, the probability that replicators of type *a* will fixate is equal to their proportion in the initial population, as in the competition between two types of Malthusian replicators. In fact, apart from a difference in time scales, the replicator Equations (3) and (28) describing these two scenarios in the deterministic limit are the same, which may explain the similarity between the finite population results.

**Figure 12:**
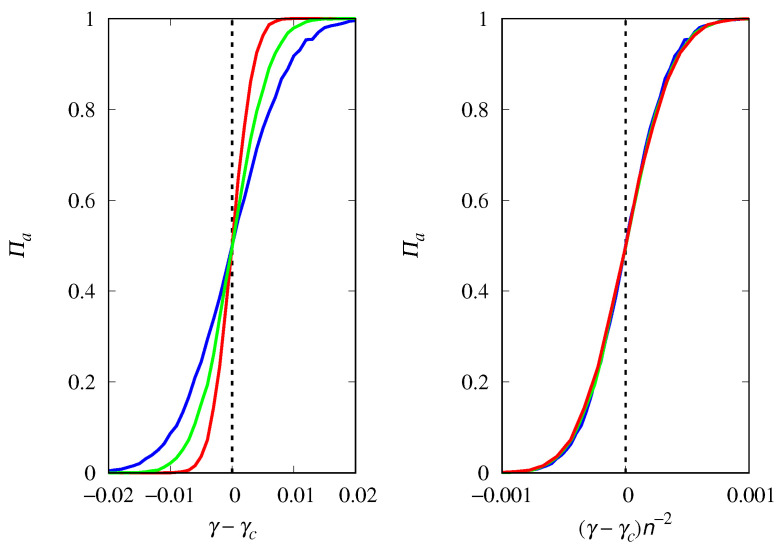
(**Left**) Probability of fixation $\Pi_{a}$ of replicators of type *a* (cooperators) as a function of the shifted variable $\gamma -\gamma_{c}$, for M=1000 and groups of size n=3 (red curve), n=4 (green curve), and n=5 (blue curve). (**Right**) $\Pi_{a}$ as a function of the scaled variable $\left(\gamma -\gamma_{c}\right)n^{-2}$. The initial condition is $x_{a}\left(0\right)=0.5$ and $\gamma_{c}=n$.

The influence of the group size *n* on the fixation probability $\Pi_{a}$ is shown in Figure 12 for a population size M=1000. The threshold is smoothed as *n* increases, and the results show that the steepness of the threshold transition decreases with $n^{-2}$. Thus, for M≫n, we can write a general scaling form for $\Pi_{a}$ near the threshold $\gamma_{c}$,

(31) $$\Pi_{a}=h\left[\left(\gamma -\gamma_{c}\right)\frac{M}{n^{2}}\right],$$

where the scaling function h(u) is such that $h\left(0\right)=x_{a}\left(0\right)$. This is an empirical equation that summarizes the data of Figure 11 and Figure 12. This way of summarizing information from data by finding properly scaled variables is the basis of the finite-size scaling technique of statistical physics [28]. Therefore, the effect of demographic noise is greatly magnified by increasing the group size *n*. This is somewhat counterintuitive, as we would normally expect to see a reduction in noise as group size increases. However, since groups are distinguished by the number of replicators of type *a*, increasing *n* actually increases the variability among group compositions, which may help demographic noise to nudge the stochastic trajectories away from the deterministic prediction.

**Figure 13:**
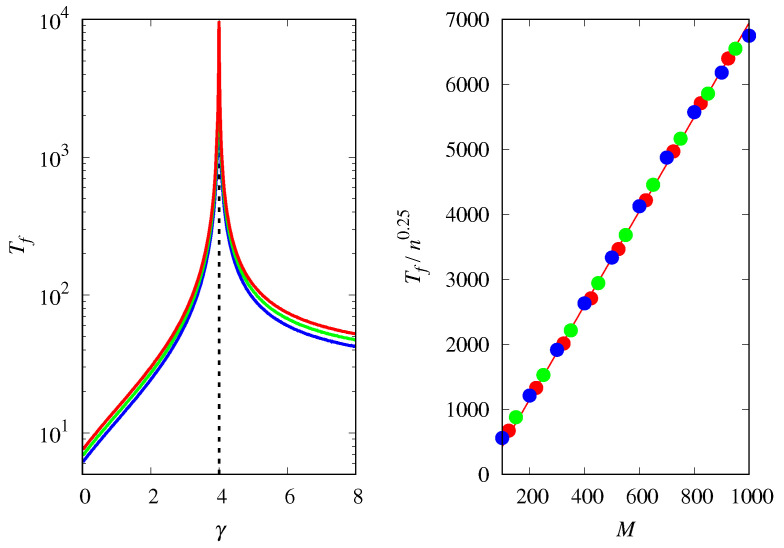
(**Left**) Mean fixation time $T_{f}$ as a function of γ for groups of size n=4 and M=1000 (red curve), M=500 (green curve), and M=250 (blue curve). The vertical dashed line indicates the threshold $\gamma_{c}=n$. (**Right**) $T_{f}/n^{0.25}$ as a function of *M* at the threshold $\gamma_{c}$ for n=3 (red symbols), n=4 (green symbols), and n=5 (blue symbols). The line is the fit $T_{f}/n^{0.25}=-298.14+7.25M$. The initial condition is $x_{a}\left(0\right)=0.5$.

Figure 13 shows how the mean fixation time $T_{f}$ is affected by the magnification factor γ, the population size *M* and the group size *n*. As indicated in Figure 10, fixation takes longer in the regime where type *a* replicators are dominant. This observation is confirmed by the results shown in the left panel of Figure 13, where we have used the logarithmic scale on the y-axis to emphasize the small differences in fixation time for the two types of replicators, which are only noticeable away from threshold. In fact, the right panel of this figure shows that the fixation time at the threshold increases linearly with *M*, but sublinearly with *n*, viz, $T_{f}∼n^{0.25}$.

## 4. Discussion

The replicator equation, which has been aptly called the “equation of life” [51], is a central component of evolutionary game theory since it governs the evolution of the frequencies of competing strategies in the limit of infinitely many players [5,31]. However, the replicator equation was used to describe chemical or prebiotic evolution, i.e., the chemical kinetics of template-directed and protein-mediated self-replicating molecules, called replicators, long before the idea of viewing the competition for building blocks or resources in general as a game [3,4]. At some point, the evolutionary game theory community and the prebiotic evolution community parted ways, and some interesting connections were lost, especially those between the dynamics of replicators temporarily confined in compartments and the public goods games. In particular, we emphasized here that the *n*-player prisoner’s dilemma, where part of the contribution to the public good is returned to the contributors themselves, representing the weak-altruism situation [30], is identical to the problem of non-specific enzyme production in the early replicator competition scenario [14]. The disconnect between these lines of research is illustrated by the parallelism of the works. For example, the effect of synergism (i.e., division of labor) in enzyme production, where the production of the enzyme requires the presence of a minimum number of enzymatic replicators in the group [52,53], is identical to a variant of the *n*-player prisoner’s dilemma where a minimum number of cooperators is needed to produce the public goods [54].

Here, we use tools developed in evolutionary game theory to revisit the population biology of the early replicators [14]. In particular, we use the stochastic algorithms that simulate the imitation (or copy) dynamics in finite population evolutionary games [26,27] to study the effect of demographic noise on the competition between replicators with two distinct characteristics. As expected, we find that demographic noise smooths out the sharp transition in the parameter space between regimes where one or the other replicator type dominates. We have not found stable coexistence between different replicator types in the competition scenarios considered here. The use of finite-size scaling allows a concise description of the fixation probability and mean fixation time near the threshold for large but finite population sizes *M*. We show that in the case of well-mixed populations of non-enzymatic (i.e., Malthusian) replicators, this probability is described by Kimura’s formula for the probability of fixation of an allele with a small selective advantage or disadvantage [25]: the sharpness of the threshold increases linearly with increasing *M*. At the threshold, where the intrinsic growth rate of the two types of replicators is equal, the mean fixation time increases linearly with *M*, consistent with Kimura’s diffusion theory. In the case of the competition between two enzymatic (i.e., hypercyclic) replicators, we find that the threshold region shrinks with $1/M^{1/2}$ and the mean fixation time at the threshold increases with lnM as *M* increases. Thus, although evolution is much faster in the case of protein-mediated replication, demographic noise is more likely to steer the stochastic dynamics toward the fixation of the replicator type that would lose the competition in the deterministic limit. We find similar results for the competition between enzymatic and non-enzymatic replicators in well-mixed populations.

A key problem in the population biology of early replicators is to explain the evolution of replicators that, in addition to template-directed replication, produce a non-specific enzyme that promotes their replication. The cost of producing the enzyme is paid as a reduction in the rate of template-directed replication. These enzymatic replicators cannot evolve in well-mixed populations because of the competition with free-riding replicators, i.e., replicators that benefit from the enzyme without paying the cost of its production. But they can evolve in the case of structured populations, where groups of *n* replicators are constantly assembled and disassembled following Wilson’s trait group formulation [45]. The fitness of replicators is determined locally within their groups, but the competition involves the entire population. This process is identical to evolutionary *n*-player games and is described in the deterministic limit by a replicator equation [46]. In particular, the evolution of enzymatic replicators requires a scenario of weak altruism, where the benefit of enzymatic replication exceeds the cost of producing the enzyme. This is a well-studied model in the deterministic limit [14,47], and we offer here a complete description of the fixation probability of the enzymatic replicators for large populations near the threshold: the sharpness of the threshold increases with *M* and decreases with $n^{-2}$. In addition, the mean fixation time at the threshold increases linearly with *M* but sublinearly with *n*. As in the case of well-mixed populations, there is no coexistence between different types of replicators: coexistence requires differential extinction of groups depending on their composition [18,19].

It seems appropriate to conclude this contribution to the study of the effects of demographic noise on the population biology of early replicators in the same way as the original paper that studied the deterministic, noiseless scenario [14], viz. by quoting Manfred Eigen in his paper that laid the foundations for a theory of the evolution of biological macromolecules [3]:

It is beyond the scope of this paper to discuss the details of the reaction mechanisms …the properties of which resemble, in many ways, social behavior.

Thus, the intersections between prebiotic evolution and public goods games have been evident from the very beginning of theoretical studies of the origin of life. It is therefore not surprising that tools developed in one field can be used in the other, as shown here.

## Data Availability

Data will be made available on request.

## Appendix A

Since the proofs in the literature (see, e.g., [26,27]) do not immediately apply to the stochastic dynamics used to model the competition between the two types of Malthusian replicators introduced in Section 2.1, we present here the proof that this dynamics leads to the replicator Equation (3) in the limit of infinitely large populations.

Assume that, at time *t*, the population is composed of A(t) replicators of type *a* and M−A(t) replicators of type *b*. In addition, let $P_{i}\left(t\right)$ be the probability that the replicator *i* is of type *a* (i.e., its intrinsic growth rate is $r_{a}$) at time *t*. The probability $P_{i}\left(t+\delta t\right)$ is given by the sum of the probabilities of the following independent and exclusive events.

(a) Replicator *i* is of type *a* at time *t*, and another replicator is selected to be challenged. The probability of this event is
  (A1) $$P_{i}\left(t\right)\times \frac{M-1}{M};$$
(b) Replicator *i* is of type *a* at time *t* and is selected to be challenged. The challenger also is of type *a*. The probability of this event is
  (A2) $$P_{i}\left(t\right)\times \frac{1}{M}\times \frac{A(t)-1}{M-1};$$
(c) Replicator *i* is of type *a* at time *t* and is selected to be challenged. The challenger is of type *b*, but fails to replace the challenged replicator. The probability of this event is
  (A3) $$P_{i}\left(t\right)\times \frac{1}{M}\times \frac{M-A(t)}{M-1}\times \left(1-\frac{r_{b}}{r_{a}+r_{b}}\right);$$
(d) Replicator *i* is of type *b* at time *t* and is selected to be challenged. The challenger is of type *a* and succeeds to replace the challenged replicator. The probability of this event is
  (A4) $$\left[1-P_{i}\left(t\right)\right]\times \frac{1}{M}\times \frac{A(t)}{M-1}\times \frac{r_{a}}{r_{a}+r_{b}}.$$

Adding up these probabilities and keeping only terms of the order of 1/M or less yields

(A5) $$P_{i}\left(t+\delta t\right)=P_{i}\left(t\right)-P_{i}\left(t\right)\frac{1}{M}\frac{M-A(t)}{M}\frac{r_{b}}{r_{a}+r_{b}}+\left[1-P_{i}\left(t\right)\right]\frac{1}{M}\frac{A(t)}{M}\frac{r_{a}}{r_{a}+r_{b}}.$$

Since $P_{i}\left(t+\delta t\right)-P_{i}\left(t\right)$ has to be proportional to δt, we have to set δt=1/M. Taking the limit M→∞ yields

(A6) $$\frac{dP_{i}}{dt}=-P_{i}\left(t\right)\left[1-x_{a}\left(t\right)\right]\frac{r_{b}}{r_{a}+r_{b}}+\left[1-P_{i}\left(t\right)\right]x_{a}\left(t\right)\frac{r_{a}}{r_{a}+r_{b}},$$

where

(A7) $$x_{a}\left(t\right)=\lim_{M\to \infty }\frac{A(t)}{M}$$

is the frequency of replicators of type *a* at time *t*. The final step in the proof is to assume that all replicators are identical in the sense that they have the same probability of being of one type or the other, i.e., $P_{i}\left(t\right)=P\left(t\right)$ for i=1,…,M. But then P(t) can be interpreted as the probability that a randomly chosen replicator is of type *a*. Thus, the law of large numbers [55] allows us to write $P\left(t\right)=x_{a}\left(t\right)$ so that Equation (A6) becomes

(A8) $$\frac{dx_{a}}{dt}=\frac{1}{r_{a}+r_{b}}\left(r_{a}-r_{b}\right)x_{a}\left(1-x_{a}\right),$$

which is the replicator Equation (3) with the time scale $\alpha =1/(r_{a}+r_{b})$.

## Appendix B

Here, we show how to integrate Equations (10) and (16) explicitly to get an analytical expression for the time *t* as a function of the frequency of replicators of type *a*, i.e., $t=t(x_{a})$. Since the final result is a graph of $x_{a}$ versus *t*, it does not matter whether we have an explicit expression $x_{a}=x_{a}\left(t\right)$ or $t=t(x_{a})$. Consider the replicator equation

(A9) $$\frac{dx_{a}}{dt}=\beta x_{a}\left(1-x_{a}\right)\left(x_{a}-\xi \right),$$

where β and ξ are parameters that can be set appropriately to recover Equations (10) and (16). It can be rewritten as

(A10) $$\frac{dx_{a}}{x_{a}\left(1-x_{a}\right)\left(x_{a}-\xi \right)}=\beta dt.$$

The right-hand side of this equation can be broken down into partial fractions, which give us

(A11) $$\frac{1}{\xi }\left[\frac{dx_{a}}{\left(x_{a}-\xi \right)}-\frac{dx_{a}}{x_{a}}\right]+\frac{1}{1-\xi }\left[\frac{dx_{a}}{\left(x_{a}-\xi \right)}+\frac{dx_{a}}{1-x_{a}}\right]=\beta dt.$$

The integrations are now easy to perform and result in

(A12) $$\frac{1}{\xi }\left[\ln\left(x_{a}-\xi \right)-\ln x_{a}\right]+\frac{1}{1-\xi }\left[\ln\left(x_{a}-\xi \right)-\ln\left(1-x_{a}\right)\right]=\beta t+C,$$

where *C* is an integration constant determined by the initial frequency $x_{a}\left(0\right)$, i.e.,

(A13) $$C=\frac{1}{\xi }\left[\ln\left(x_{a}\left(0\right)-\xi \right)-\ln x_{a}\left(0\right)\right]+\frac{1}{1-\xi }\left[\ln\left(x_{a}\left(0\right)-\xi \right)-\ln\left(1-x_{a}\left(0\right)\right)\right].$$

Finally, inserting this value of *C* in Equation (A12) and rearranging the terms yield

(A14) $$\beta t=\frac{1}{\xi (1-\xi )}\ln\left[\frac{x_{a}-\xi }{x_{a}\left(0\right)-\xi }\right]-\frac{1}{\xi }\ln\left[\frac{x_{a}}{x_{a}\left(0\right)}\right]-\frac{1}{1-\xi }\ln\left[\frac{1-x_{a}}{1-x_{a}\left(0\right)}\right].$$
